# Modern Sociology

**Published:** 1900-05-05

**Authors:** 


### Modern Sociology.

### THE LEGALISATION OF THEFT.

We take the following from the *Globe* of March 13th:—"The Committee of the French Chamber of Deputies has adopted a Bill providing that absolute destitution shall be held a sufficient excuse for theft of food."

Our friends across the Channel are nothing if not logical. Occasionally—very seldom, we fancy—a man or woman who has hitherto been honest steals food under stress of hunger; therefore they ordain that the plea of hunger shall be accepted as a sufficient reason for stealing food. This is sentimentalism with a vengeance. We have sentimentalists in England also, who sympathise with the poor dear thief; but on the whole our sympathies are, wisely, with the owner of the stolen property, who in all probability has bought and paid for it honestly, and must bear the brunt of the theft, even if the thief is punished. There has never been any legislation, so far as we know, to recoup the person robbed for his loss, if he cannot make the thief do so! In cases where it can be shown that sheer destitution has led to the crime our judges as a rule impose only a slight penalty, and there are usually plenty of charitable persons who are willing to do what they can to prevent the thief from ever being again put into a position of such temptation. But we have never pretended that the crime, though circumstances made it comprehensible, was not a crime—an offence against society which deserved punishment.

To what strange consequences would such legislation as that which has obtained acceptance in France lead us! One might easily work it out to a *reductio ad absurdum*. A man needs more than food; decency and comfort demand a certain amount of clothing. If the boots of a tramp are worn out, or if—an opportunity for the legalised theft of food not being handy—he has pawned his coat to procure it, what is there to hinder him from claiming legislative sanction for replenishing his wardrobe at someone else's expense? Necessity has no law, or, rather, necessity has now an obliging law of its own. Nor can we, in this climate at least, do without shelter for great part of the year. What then is to hinder our impecunious friend, under the plea of necessity, from demanding the unwilling hospitality of our spare bedroom? It is all on a par; the idea all through is that a man, merely because he is necessitous, has a right to something which other people have acquired lawfully, and which they are not willing to give him of their own accord. Well, the least that the Government can do that permits such action is to pass a law providing for the compensation of the robbed. But perhaps our French friends would plead that they are acting on the maxim that "property is theft," and merely arranging that those who have not managed to do their thieving in one way should be allowed to do it in another.

But the fact that such legislation seems to belong rather to the sphere of comic opera than to that of real life should not blind us to its dangerous tendencies. It is part of a general inclination to ignore conduct as a factor in well-being, and those fundamental virtues on which conduct depends. It is easy to say that poverty is a misfortune and not a crime, but it is very seldom that honest poverty sinks to absolute destitution—and even then it doesn't steal. It is more likely to starve instead. Where pride is not so keen there are plenty of institutions in every civilised country whereby respectable poverty is relieved. If people reach the point of absolute starvation before applying to these it is their own fault. It may be safely said that no one need die of starvation, nor steal to avoid it. What then is the use of special legislation for the thief? It can only make him feel—it is meant to make him feel—that he is guilty of no fault. His destitution may be due to his own improvidence or vice. What of that? He is destitute, and therefore has a right to help himself to what he wants. Is this the way to make him strive to be better? Perhaps that might be hopeless in any case; so let us ask instead, is this the way to keep others from those faults which lead to destitution? Such legislation not only destroys such influence as may exist to keep the destitute man in the paths of honesty, but it lessens that fear of destitution which keeps many men not only honest, but industrious. Why should one do work that is hard and uncongenial when, at the worst, one can always steal without thereby incurring either punishment or reproach? Without claiming to be much of a prophet, one may safely say that if the Bill for the legalisation of theft becomes law, France will witness not only a great increase of theft, for which the plea of destitution will be urged, but a great falling off in the industry of the poorer classes of workers.

We do not wish to preclude an appeal *ad misericordiam*, when it can be shown that the temptation was strong, and the means of legitimate relief difficult to obtain. But such cases must be judged on their individual merits, and whatever consideration they may receive should not be allowed to hide the fact that the theft was a crime. The common cry that there are thieves in high places who are beyond the reach of the law should have no bearing on our judgment. Even if the accusation were far more solidly founded than it has ever yet been shown to be, it would be no excuse for condoning an admitted crime. Let us extend the law so as to capture and bring to justice all thieves, in whatever way they do their thieving, but to whittle away the guilt of theft or any other crime is to lessen the safeguards which protect the community from being over-run by the unscrupulous and ill-doing. The French have a reputation for informally accepting "extenuating circumstances" in cases of murder to a too considerable extent. They are also popularly supposed to hold in very light esteem that denunciation of unlawful passion which we find in the Decalogue. We have recently had reason to suppose that they take no account of that commandment which forbids a man to give false witness against his neighbour, and now we find them desirous of abrogating the commandment which says definitely "Thou shalt not steal." They evidently have no regard for Moses and his law. But that law has the sanction of God and the experience of man for thousands of years to support it, and woe unto man or nation that ignores or trifles with it, for it is the foundation on which civilised society rests!

